# Size selectivity in antibiofilm activity of 3-(diphenylphosphino)propanoic acid coated gold nanomaterials against Gram-positive *Staphylococcus aureus* and *Streptococcus mutans*

**DOI:** 10.1186/s13568-017-0515-x

**Published:** 2017-11-21

**Authors:** Dania Ahmed, Ayaz Anwar, Anum Khalid Khan, Ayaz Ahmed, Muhammad Raza Shah, Naveed Ahmed Khan

**Affiliations:** 1 H.E.J. Research Institute of Chemistry, International Center for Chemical and Biological Sciences, University of Karachi, Karachi, 75270 Pakistan; 2 Department of Biological Sciences, School of Science and Technology, Sunway University, 47500 Subang Jaya, Selangor Malaysia; 3 Dr. Panjwani Center for Molecular Medicine and Drug Research, International Center for Chemical and Biological Sciences, University of Karachi, Karachi, 75270 Pakistan

**Keywords:** Gold nanomaterials, 3-(diphenylphosphino)propanoic acid, Antibiofilm activity, *S. aureus*, *S. mutans*, AFM

## Abstract

Biofilm formation by pathogenic bacteria is one of the major threats in hospital related infections, hence inhibiting and eradicating biofilms has become a primary target for developing new anti-infection approaches. The present study was aimed to develop novel antibiofilm agents against two Gram-positive bacteria; *Staphylococcus aureus* (ATCC 43300) and *Streptococcus mutans* (ATCC 25175) using gold nanomaterials conjugated with 3-(diphenylphosphino)propionic acid (Au-LPa). Gold nanomaterials with different sizes as 2–3 nm small and 9–90 nm (50 nm average size) large were stabilized by LPa via different chemical synthetic strategies. The nanomaterials were fully characterized using atomic force microscope (AFM), transmission electron microscope, ultraviolet–visible absorption spectroscopy, and Fourier transformation infrared spectroscopy. Antibiofilm activity of Au-LPa nanomaterials was tested using LPa alone, Au-LPa and unprotected gold nanomaterials against the both biofilm-producing bacteria. The results showed that LPa alone did not inhibit biofilm formation to a significant extent below 0.025 mM, while conjugation with gold nanomaterials displayed manifold enhanced antibiofilm potential against both strains. Moreover, it was also observed that the antibiofilm potency of the Au-LPa nanomaterials varies with size variations of nanomaterials. AFM analysis of biofilms further complemented the assay results and provided morphological aspects of the antibiofilm action of Au-LPa nanomaterials.

## Introduction

Nanotechnology offers exceptional approaches towards controlling a variety of pivotal biological processes and is believed to have an influence on several biological systems since they also occur at the nanometer dimension. Applications of nanotechnology in medicine are immense and have paved the way for the development of new and effective medical treatments (i.e., nanomedicine) (Emerich and Thanos 2003). The monitoring of size and structure at the nanoscale ensures advantages of nano-medicines over conventional therapies due to targeted drug delivery, enhanced bioavailability and bio-conjugation (Geethalakshmi and Sarada 2013).

Microorganisms in general possess an extraordinary ability to settle and survive in precisely programmed regions of hosts and cause malfunction in biological routine. On the other hand, bacterial infections due to emerging multidrug-resistant (MDR) and the lack of development of new and effective drugs represent a devastating problem in healthcare. MDR bacteria contribute to morbidity and mortality rates in many of the common infectious diseases (Franci et al. 2015). *Staphylococcus aureus* is known to exfoliate the epidermal layers and localizes within the skin causing wound infections, while it can also penetrate lungs, bloodstream, joints, and bones in some extreme cases (Chwalibog et al. 2010). Whereas, production of extracellular polysaccharides and acids from dietary sugar and adhesion to dental enamel are amongst the most common clinically important features of *Streptococcus mutans* that causes oral cavities and tooth decay (Choi et al. 2001).

Biofilms are microbes bound together in an extracellular matrix of polymeric substances. These are simply characterized by irreversibly attached bacterial cells to any surface or to each other (Davey and O’toole 2000). The morphology and physiological characteristics of biofilm producing microorganisms adapt resistance up to 1000 times against antimicrobial agents in contrast to their planktonic counterparts (Mah et al. 2003). Various pathogens tend to produce biofilms on food and/or storage surfaces, while some pathogenic bacteria such as Methicillin resistant *Staphylococcus aureus* (MRSA), *Escherichia coli*, *Klebsiella pneumoniae* and *Pseudomonas* species contribute majorly in nosocomial infections in hospitals on implanted medical devices, especially catheter related urinary tract infections (CAUTIs) (Bryers 2008). According to reports from national institute of health (NIH), biofilm infections are suggested to exceed by 60% in the developed world alone (Lewis 2001). Therefore, it is imperative to develop novel antibiofilm agents for inhibition and eradication of already formed biofilms. Recently, metal and metal oxide nanoparticles have been found to be a useful alternate to inhibit microbial growth and prevent biofilm formation (Dhandapani et al. 2012; Mu et al. 2016). Several antibiotics coated with metallic nanoparticles have recently been reported to produce interesting antibiofilm and antibacterial effects (Ahmed et al. 2016; Singh et al. 2014).

Inorganic nanoparticles are well known to interact with microorganisms and thus act as antibacterial and antifungal agents (Rai et al. 2009; Taglietti et al. 2014). Gold nanoparticles have presented tremendous applications in almost every field of science especially in biology. Grace and Pandian have reported the use of gold nanoparticles as a carrier of aminoglycosidic antibiotics like streptomycin, gentamycin and neomycin. Their results demonstrated high efficacy of gold nanocomposites of these drugs against various Gram-negative and Gram-positive bacteria (Grace and Pandian 2007). Nanoparticles interaction with bacteria is dependent on various factors including the size, morphology and coating of the nanoparticles. The antibacterial activity of the nanoparticles has been found to alter vastly with modification in size of nanoparticles (Boda et al. 2015; Martinez-Castanon et al. 2008).

The aim of this study was to determine the influence of particle size of gold nanomaterials capped with 3-(diphenylphosphino)propionic acid on bacterial biofilms of *S. aureus* (ATCC 43300) and *S. mutans* (ATCC 25175). AFM studies were also carried out to study the morphological changes occurred after treatment of small and large size gold nanomaterials.

## Materials and methods

All chemicals and solvents were used as purchased without any purification or pretreatment unless stated otherwise. Tetrachloroauric acid (HAuCl_4_), chloro(triphenylphosphine)gold(I) {Au(PPh_3_)Cl}, borane-*t*-butylamine complex (BTBC), sodium borohydride (NaBH_4_) and ligand 3-(diphenylphosphino)propionic acid (LPa) were purchased from Sigma-Aldrich (St. Louis, USA). All the solvents used were either HPLC or analytical grade, and were de-aerated according to standard protocols prior to use.

### Synthesis of Au-LPa large

Synthesis of Au-LPa nanoparticles involves the reduction of HAuCl_4_ by NaBH_4_ in the presence of LPa as stabilizer. Briefly, NaBH_4_ (0.015 mg in 0.1 mL deionized water) was added to a vigorously stirred HAuCl_4_ solution (3.94 mg in 95 mL deionized water) and LPa (0.143 mg in 5 mL deionized water and 0.5 mL of methanol) at room temperature. A reddish pink color solution was formed, showing a surface plasmon resonance (SPR) absorption peak at 524 nm confirms the formation of gold nanoparticle. The colloidal suspension was subjected to centrifugation at 10,000 rpm to separate the nanoparticles from unreacted reagents especially toxic reducing agent and salt produced. Nanoparticles pellet was obtained as a result of centrifugation and was then re-suspended in water.

### Synthesis of Au-LPa small

The synthetic procedure used for Au-LPa nanoclusters was as previously described (Woodworth et al. 2016). Precisely, the mixture of 1 mol equiv. of Au(PPh_3_)Cl and phosphine ligand LPa was taken in a de-aerated methanol in an oven dried Schlenk flask under argon atmosphere. Vigorous stirring was continued and BTBC was added with 5 mol equiv. as compared to the gold precursor. Following this, the reaction mixture turned to orange within 1 h. Further stirring was continued for 4 h to make sure the reaction was completed. Products obtained were characterized by UV–visible spectrophotometry, and TEM. Nanoclusters were also subjected to the same post synthesis treatment as described above.

### Synthesis of gold nanomaterials control (unprotected)

Both small and large gold nanomaterials alone used as controls were synthesized as described above and at similar concentrations in the absence of stabilizing agent LPa.

### Biofilm inhibition potential of compounds

Antibiofilm activities of the Au-LPa nanomaterials were screened against the two bacterial strains using crystal violet method as described previously (Ahmed et al. 2016). Biofilm inhibition was evaluated by using following equation:

$$ \% {\text{ biofilm inhibition }} = \left\{ {\left( {{\text{O.D. in control }}-{\text{O.D. of test}}}\right)/{\text{O.D. in control}}} \right\}\times 100 $$

### Biofilms imaging via atomic force microscope (AFM)

The antibiofilm potential of test samples against *S. mutans* (ATCC 25175) and *S. aureus* (ATCC 43300) was further studied by atomic force microscopic images analysis. Nanomaterials at concentrations 0.0025 mM were used for AFM analysis. Biofilm formation was carried out in 24-well plate containing 8 mm circular cover slips. After incubation the cover slips were heat fixed and scanned by atomic force microscope (Agilent 5500). ACAFM mode was used with triangular shape silicon nitride soft cantilever (Veeco, model MLCT-AUHW) for AFM analysis.

## Results

### Characterization of Au-LPa nanomaterials

**Fig. 1:**
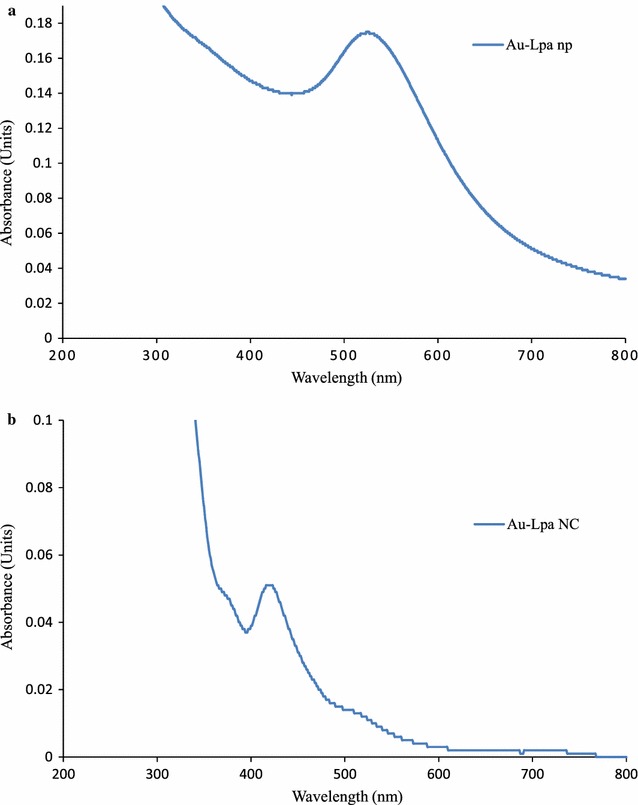
UV–visible spectrum of Au-LPa nanomaterials. **a** Au-LPa large show surface plasmon resonance band at 524 nm. **b** Au-LPa small show characteristic absorption signal at 418 nm

Gold nanomaterials formation by reduction of metal ion in the presence of LPa was determined using UV–visible spectroscopy (Fig. 1). Au-LPa nanoparticles revealed absorbance maxima at 524 nm which is the characteristic absorbance for un-aggregated colloidal suspension of gold nanoparticles. While the orange color and absorption maxima at lower wavelength for gold nanomaterials correspond to the smaller particle size, particularly less than 2 nm which is in agreement with our previously reported method for the size selective synthesis of gold nanoclusters (Woodworth et al. 2016).

### Size determination of the Au-LPa nanomaterials

**Fig. 2:**
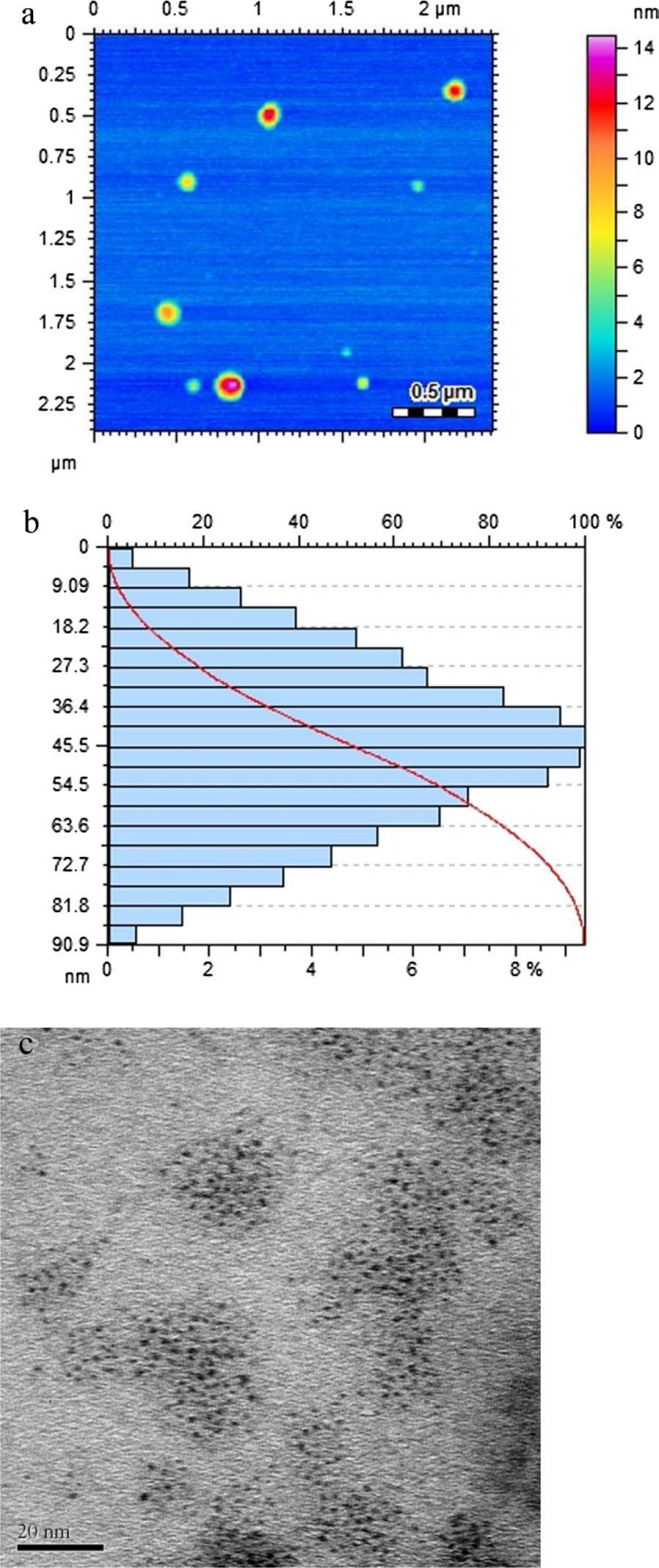
Size and morphology of Au-LPa nanomaterials. **a** AFM image of Au-LPa large. **b** Size distribution histogram of Au-LPa large. **c** TEM image of Au-LPa small

Atomic force microscopic studies of Au-LPa large were performed through tapping mode atomic force microscopy in order to determine the particle size and morphology of these nanoparticles. As depicted in Fig. 2, the nanoparticles were poly-dispersed, spherical in shape and their size ranged from 9 to 90 nm with an average size of 50 nm. While Au-LPa small were prepared by our previously optimized size selective synthesis ensuring the particle size in the range of 2–3 nm as shown in the TEM image (Fig. 2c).

### FT-IR analysis

**Fig. 3:**
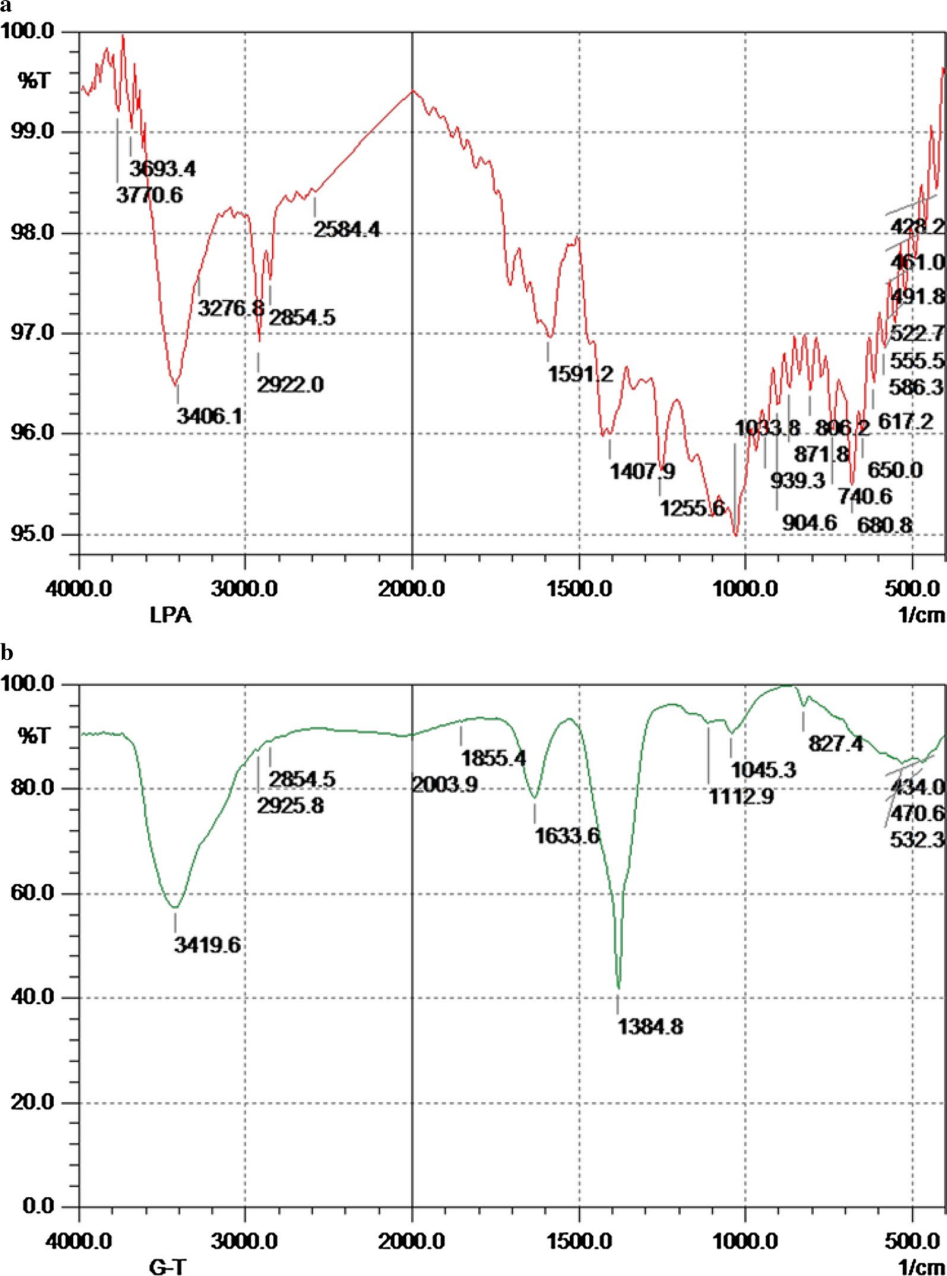
FT-IR spectra of (**a**) ligand LPa, (**b**) Au-LPa large nanomaterials

FT-IR analysis of Au-LPa and LPa alone was also carried out in order to determine the functionalities found in ligand that are responsible for the stabilization of nanomaterials (Fig. 3). Both the phosphine and carboxylic acid functionalities were found to be responsible for capping gold nanomaterials. The FT-IR spectrum of LPa shows four distinct absorption bands that appear at 3406 cm^−1^ which corresponds to the OH group, another appears at 2922 cm^−1^ because of CH stretching vibration. Carbonyl stretching vibration appears at 1720 cm^−1^ and Phosphorous Carbon bond vibration appears at 1407 cm^−1^. Following nanoparticles formation, distinct changes were visible in the FT-IR spectrum. An absorption band appearing at 3406 cm^−1^ shifted to 3419 cm^−1^, and a bend appeared due to carbonyl group vibration moved to 1633 cm^−1^. Bands appearing due to phosphorus and carbon bond stretching shifted to 1384 cm^−1^ and became more intense which suggests the stabilizing interaction of phosphine group with gold nanomaterials. These results are also consistent with previous reports, as phosphines and hydroxyl groups have been extensively used for metal nanoparticles preparation with narrow size distribution and higher stability (Wu et al. 2006).

### Stability of Au-LPa nanomaterials

**Fig. 4:**
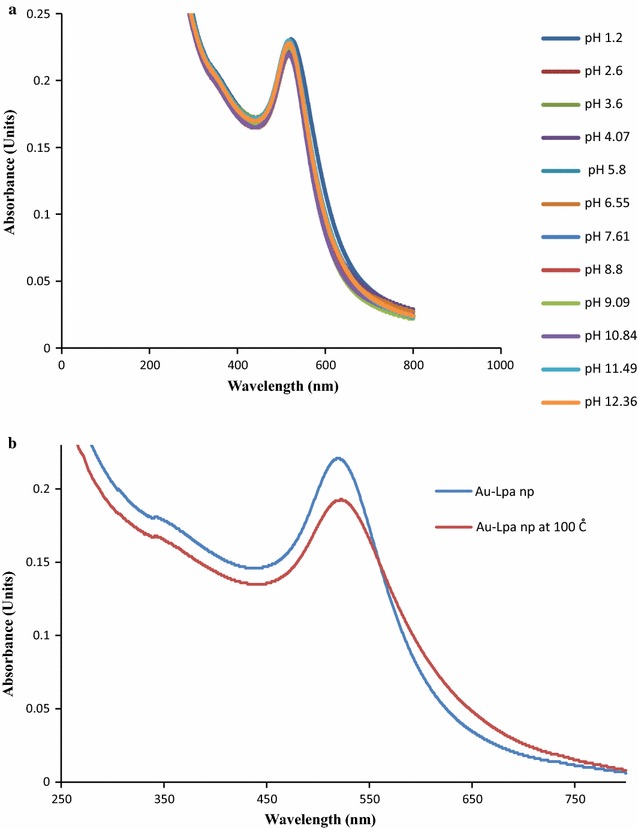
**a** UV–Visible spectra showing no effect of pH on Au-LPa nanomaterials stability. **b** UV–visible spectrum for temperature tolerance of Au-LPa

pH effects on the stability of Au-LPa were also evaluated to test the robustness of the synthetic procedure. Figure 4a shows that nanoparticles were found to be stable in a pH range from 1 to 10 as evident from the UV–visible spectra since there is no effect of change in pH on nanoparticle absorption intensity as well as on its position and no aggregation was noticed in the whole pH range.

Heat effects on the stability of Au-LPa were also evaluated as shown in Fig. 4b, and it was found that nanoparticles were stable upon heating up to 100 °C. However, a slight change in peak intensity of the nanoparticles was observed while the peak position remains the same with a little peak broadening which may correspond to a tiny proportion of particle aggregation. Hence Au-LPa nanomaterials were found to tolerate the alteration in the pH and temperature conditions, which suggests that they are quite stable and can be utilized in a vast variety of applications.

### Antibiofilm activity

**Fig. 5:**
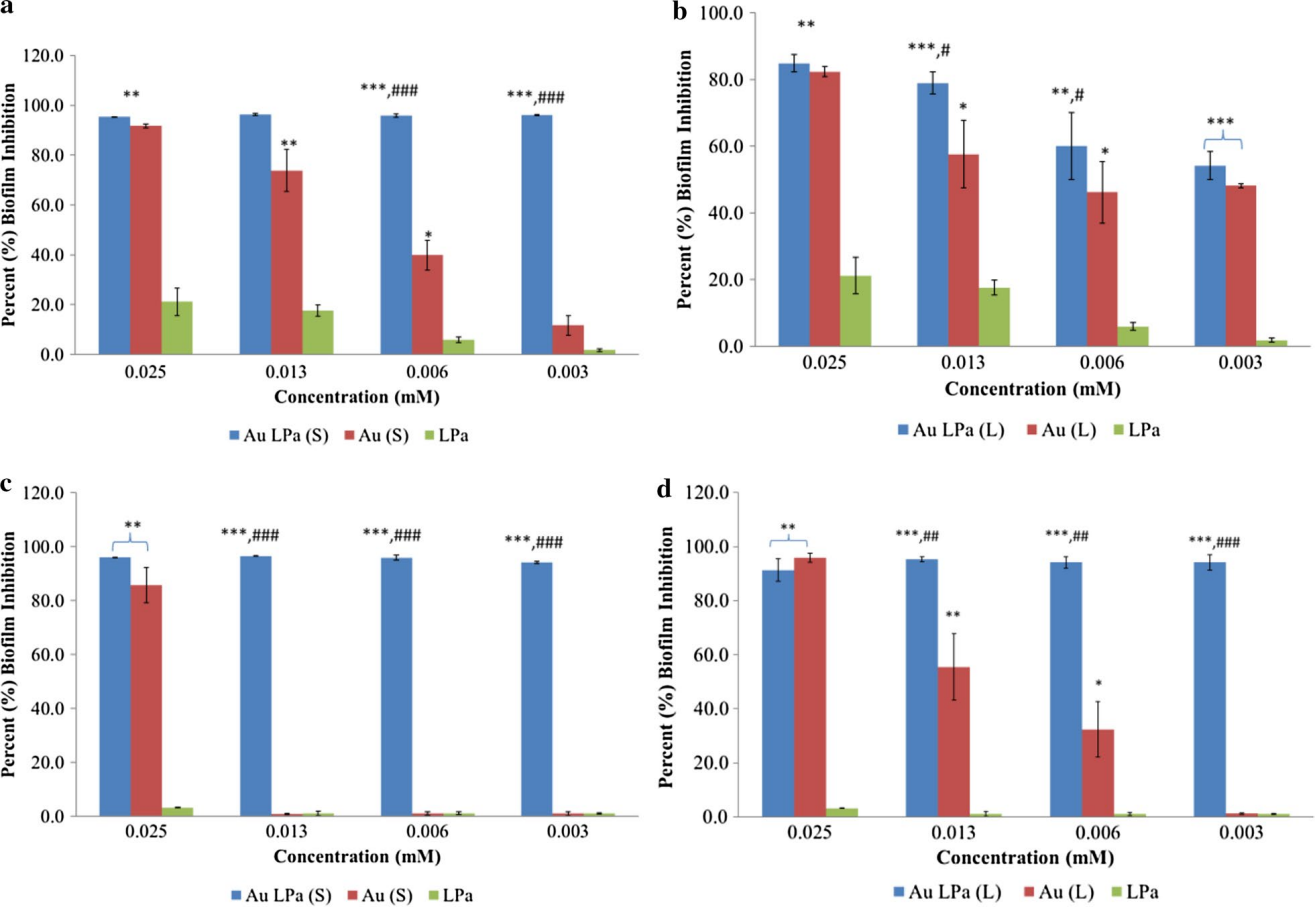
Percent biofilm inhibition of Au-LPa (small and large size) against (**a**, **b**) *S. aureus*; (**c**, **d**) *S. mutans*. Statistical significance of the data was represented as *p < 0.05, **p < 0.01 and ***p < 0.001. Whereas * represent significance as compared to LPa and # represent as compared to gold nanomaterials alone (small or large size)

% inhibition was determined for LPa solution and Au-LPa large and small against *S. aureus* (ATCC 43300) and *S. mutans* (ATCC 25175) biofilms using the crystal violet method. The biofilms were treated with four concentrations of all test samples at a level of 0.025, 0.0125, 0.00625, 0.003125 mM for 24 h. Results of the antibiofilm assay are presented in Fig. 5. Organic LPa ligand did not exert significant antibiofilm activity, however interestingly when conjugated with gold nanomaterials, significant effects were observed in antibiofilm potential. Moreover, Au-LPa small were found to be more efficient in inhibiting the biofilms of both tested Gram-positive bacteria as compared to their large counterparts. Proper controls for both small and large gold nanomaterials were also tested to negate false positive results, but both controls are found to be inactive or only slightly active at similar concentrations as compared to Au-LPa.

### Atomic force microscopy analysis for the imaging of biofilms

**Fig. 6:**
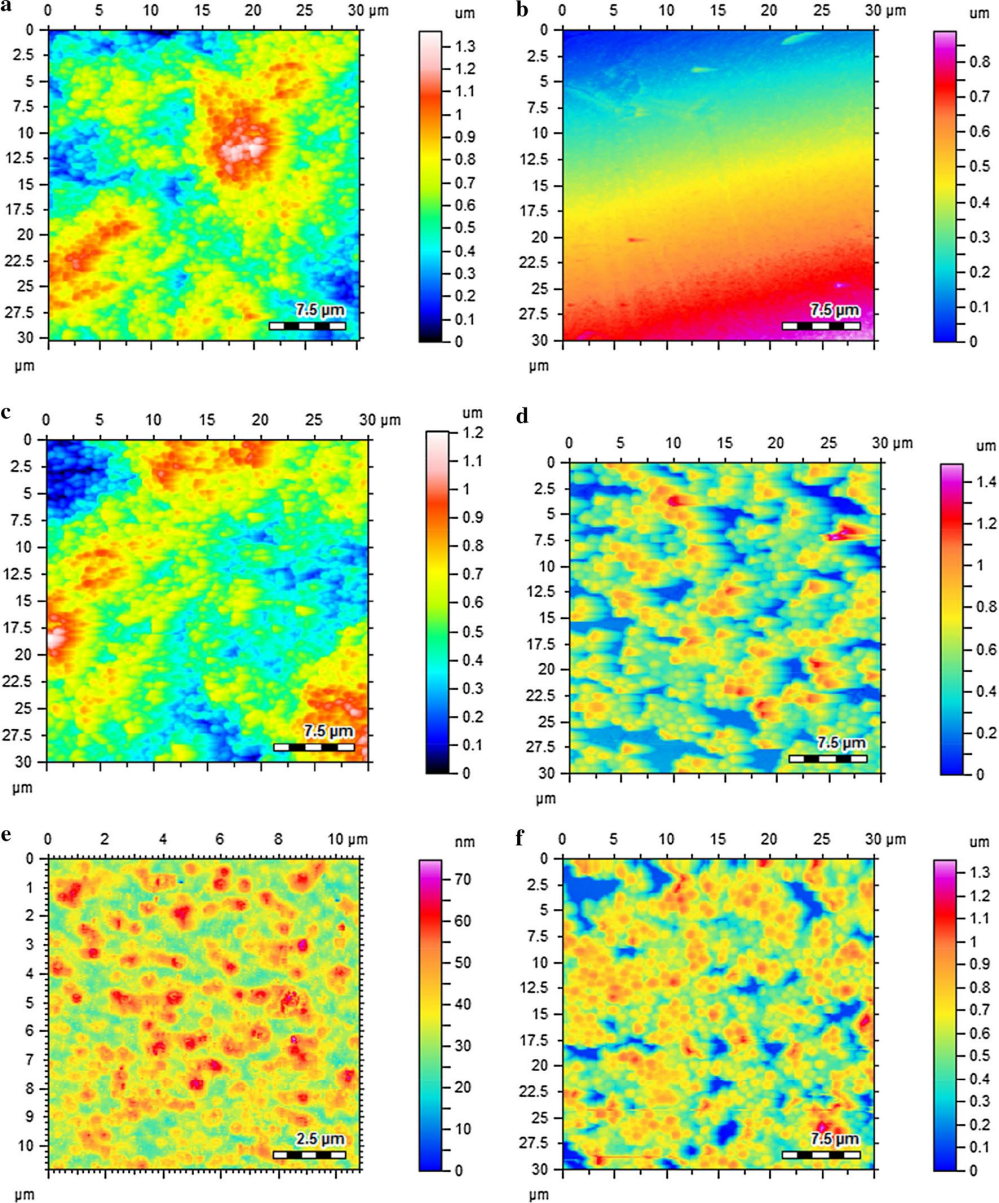
AFM topographic images of *S. aureus* biofilms; **a** biofilm untreated; **b** Au-LPa small treated; **c** Au-LPa large treated; **d** gold nanomaterials large control treated; **e** gold nanomaterials small control treated; **f** LPa alone treated

**Fig. 7:**
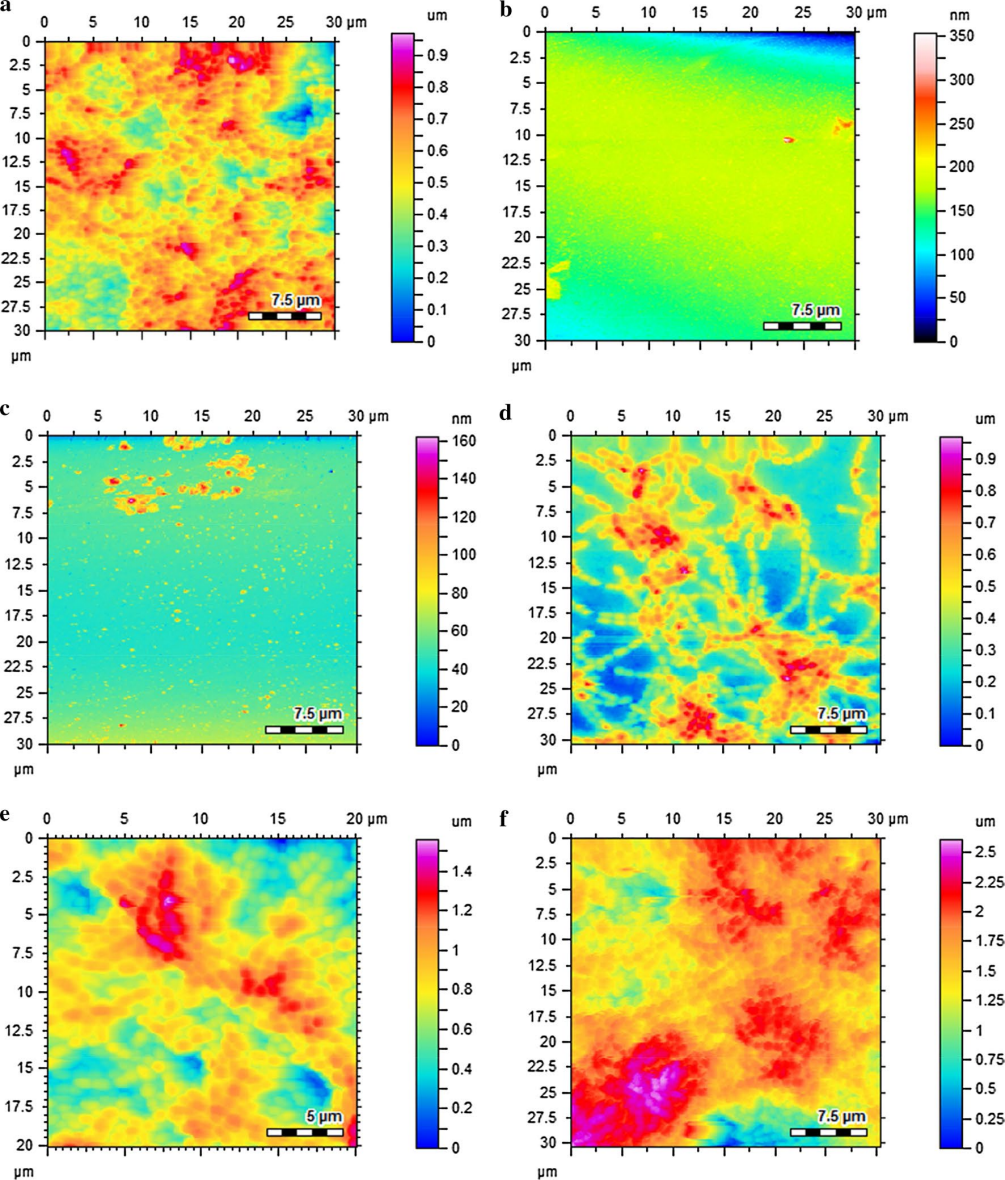
AFM topographic images of *S. mutans* biofilms; **a** biofilm untreated; **b** Au-LPa small treated; **c** Au-LPa large treated; **d** gold nanomaterials large control treated; **e** gold nanomaterials small control treated; **f** LPa alone treated

Biofilm inhibitory potential of the synthesized gold nanomaterials (small and large) of LPa was further complimented with atomic force microscopic analysis. Biofilm inhibition was observed after treating the cells with 0.0025 mM concentration of the synthesized nanoparticle, LPa, gold nanomaterials alone (small and large) and compared with controls. In the case of *S. aureus* Au-LPa small completely inhibited biofilm formation, Au-LPa large showed partial inhibition whereas gold nanomaterials (small and large) and LPa alone failed to inhibit biofilm formation (Fig. 6a–f). On the contrary, Au-LPa small and large completely inhibited the biofilm formation of *S. mutans* as compared to gold nanomaterials (small and large) and LPa alone (Fig. 7a–f).

## Discussion

After reduction of gold salts with corresponding reducing agents in the presence of LPa, Au-LPa nanomaterials were fully characterized by UV–visible, FT-IR, AFM, and TEM. Both large and small Au-LPa nanomaterials revealed characteristic surface plasmon resonance band corresponding to their sizes as supported by previous literature.

After the successful synthesis and characterization of both Au-LPa nanomaterials, these were subjected to antibiofilms assay via the crystal violet method against *S. aureus* and *S. mutans*. The results suggest that LPa alone did not inhibit the formation of both bacteria biofilms to a significant level at the tested concentrations. In particular, it had negative effect on the inhibition of *S. mutans* biofilms, while minimal positive effects on *S. aureus* biofilms were observed (21.18% inhibition only at high dose of 0.025 mM). Au-LPa nanomaterials on the other hand showed high tendency to inhibit the biofilm formation of both bacteria. Au-LPa small displayed 95–96% inhibition against both bacteria even at low concentration (0.003125 mM), and had no change in increasing the sample’s concentration. Contrary to Au-LPa small, Au-LPa large inhibited *S. mutans* to great extent even at lower concentrations but showed dose dependent response towards inhibiting *S. aureus* biofilms (54–84% inhibition at 0.003125–0.025 mM). To nullify the false positive results from Au-LPa nanomaterials, effect of gold controls (unprotected) was also evaluated by similar protocol and parameters. Both gold nanomaterials controls were freshly synthesized as described in “Materials and methods” section and were used without any pretreatment or proper characterization. Both gold nanomaterials controls are found to be ineffective at the concentration levels where Au-LPa nanomaterials showed significant inhibition, however at higher doses both controls showed positive % inhibition against both bacteria. These results can be attributed towards the toxicity of borane reducing agents whose concentration is much higher in the control samples at higher doses, since they were used as prepared. Hence such an effect was expected and completely justifiable.

The higher activity and lower selectivity of Au-LPa small as compared to Au-LPa large may be attributed to the impact of smaller size. Since the smaller size of nanoclusters guarantees the delivery of therapeutic agent inside the cells by overcoming the membrane barriers more effectively and as a result causes better activity (Martinez-Castanon et al. 2008).

AFM analysis of the antibiofilms assay further provided a clear picture of the mode of action of these test samples. After the biofilm formation, the AFM images of control samples indicated prominent and integrated biofilm surface topology of both the tested bacterial strains, i.e. *S. aureus*, *S. mutans* (Figs. 6a, 7a respectively). Figures 6b and 7b shows that the biofilms of both bacteria treated with Au-LPa small were completely diminished as was expected from the high % inhibition, hence no signs of bacteria were found in the Au-LPa small treated samples. The Au-LPa large treated samples displayed reduced biofilm as compared to controls, but significant presence of partially depleted biofilm can be observed in Fig. 6c for *S. aureus* as large nanomaterials have less efficiency to penetrate the compact biofilm. However, Au-LPa large selectively disintegrated *S. mutans* biofilms as compared to *S. aureus* (Fig. 7c), which was forecasted in the assay results (Fig. 5). It is suggested in the recent literature that antibiofilms activity of nanoparticles might be the result of nanoparticle approach the biofilm resident through water channel (Stewart 2003). Biofilms samples were then also treated with the unprotected gold nanomaterials (both large and small) to check their antibiofilm effect and validate the results of Au-LPa nanomaterials. Figures 6d, e and 7d, e represent gold controls treated biofilm images, and it is clearly evident from these images that at lower concentrations gold controls have no antibiofilm potential. Same was the case with LPa alone, as it induced no damages to the compactness of biofilms. So based on above discussion it is safe to conclude that the conjugation of gold enhanced the antibiofilm efficacy of LPa, while small Au-LPa nanomaterials are found to be more effective against both Gram-positive bacteria biofilms as compared to larger nanoparticles.

Since the potential of any antimicrobial agent to permeate biofilms and to disperse it is of great importance, and nanomaterials have provided a great deal of feasibility to overcome this problem more recently. Thus, our results demonstrate that Au-LPa nanomaterials can be a useful candidate to disrupt Gram-positive bacteria biofilms with several advantages over the antibiotics approach. Phosphines are a newer class of compounds which are not being used quite extensively for antibacterial purpose, hence there is less chance for bacterial resistance. However, phosphine gas has been used in fumigation for pest control purposes due to its known toxicity.

In conclusion, gold nanomaterials of different sizes (2–3 nm and 50 nm) coated with LPa were prepared via two different chemical methodologies. Au-LPa nanomaterials showed enhanced in vitro inhibition of biofilms of Gram-positive bacteria *S. aureus* (ATCC 43300) and *S. mutans* (ATCC 25175) as compared to the same concentration of LPa alone (0.0025 mM). This clearly indicates that gold nanomaterials conjugation with labile phosphine ligands can display antibiofilm effects. The results of the antibiofilm assay and AFM analysis demonstrated that Au-LPa small nanomaterials inhibited the biofilms more efficiently in comparison to Au-LPa large. Due to the important proposition of Gram-positive bacteria biofilms in human pathogenesis, and their antibacterial resistance, this method provides a useful candidate of interest for the applications in nanobiotechnology. However, more studies including evaluation of the toxicity profile of Au-LPa nanomaterials and their mechanism of action are our future plans.

## Abbreviations

LPa: 3-(diphenylphosphino)propionic acid
Au-LPa: gold nanomaterials conjugated with 3-(diphenylphosphino)propionic acid
Au (S): gold nanoparticles small
Au (L): gold nanoparticles large
AFM: atomic force microscope
TEM: transmission electron microscope
UV–Vis: ultraviolet–visible
FT-IR: Fourier transform infrared
BTBC: borane-*t*-butylamine complex
